# Characterization of porcine dendritic cell response to *Streptococcus suis*

**DOI:** 10.1186/1297-9716-42-72

**Published:** 2011-06-02

**Authors:** Marie-Pier Lecours, Mariela Segura, Claude Lachance, Tufaria Mussa, Charles Surprenant, Maria Montoya, Marcelo Gottschalk

**Affiliations:** 1 Groupe de Recherche sur les Maladies Infectieuses du Porc and Centre de Recherche en Infectiologie Porcine, Faculté de Médecine Vétérinaire, Université de Montréal, St-Hyacinthe, Québec, J2S 2M2, Canada; 2 Centre de Recerca en Sanitat Animal (CReSA), UAB-IRTA, Campus de la UAB, Bellaterra, Barcelona, Spain; 3 F. Ménard, 251 Route 235, Ange Gardien, Québec, Canada; 4 Institut de Recerca i Tecnologia Agroalimentàries (IRTA), Barcelona, Spain

## Abstract

*Streptococcus suis *is a major swine pathogen and important zoonotic agent causing mainly septicemia and meningitis. However, the mechanisms involved in host innate and adaptive immune responses toward *S. suis *as well as the mechanisms used by *S. suis *to subvert these responses are unknown. Here, and for the first time, the ability of *S. suis *to interact with bone marrow-derived swine dendritic cells (DCs) was evaluated. In addition, the role of *S. suis *capsular polysaccharide in modulation of DC functions was also assessed. Well encapsulated *S. suis *was relatively resistant to phagocytosis, but it increased the relative expression of Toll-like receptors 2 and 6 and triggered the release of several cytokines by DCs, including IL-1β, IL-6, IL-8, IL-12p40 and TNF-α. The capsular polysaccharide was shown to interfere with DC phagocytosis; however, once internalized, *S. suis *was readily destroyed by DCs independently of the presence of the capsular polysaccharide. Cell wall components were mainly responsible for DC activation, since the capsular polysaccharide-negative mutant induced higher cytokine levels than the wild-type strain. The capsular polysaccharide also interfered with the expression of the co-stimulatory molecules CD80/86 and MHC-II on DCs. To conclude, our results show for the first time that *S. suis *interacts with swine origin DCs and suggest that these cells might play a role in the development of host innate and adaptive immunity during an infection with *S. suis *serotype 2.

## Introduction

*Streptococcus suis *is a major swine pathogen associated mainly with meningitis, although other pathologies have also been described such as septicemia with sudden death, endocarditis, arthritis, and pneumonia [1]. Among 35 serotypes described, serotype 2 is considered the most virulent and the most frequently isolated from both diseased pigs and humans. Consequently, most studies on virulence factors and the pathogenesis of infection have been carried out with this serotype [2]. Until recently, *S. suis *disease in humans has been considered as rare and only affecting people working with pigs or pork by-products. However, with a rising incidence in humans over the last years, *S. suis *is now considered as an important emerging zoonotic agent, especially in Asian countries, where *S. suis *has recently been identified as the leading cause of adult meningitis in Vietnam, the second in Thailand, and the third in Hong Kong. In 2005, an important outbreak occurred in China and resulted in 200 human cases with a fatality rate near 20% [1]. In humans, *S. suis *is mainly responsible for meningitis, septicemia and streptococcal toxic shock-like syndrome [1,3,4].

Despite the increasing number of studies, the pathogenesis of the *S. suis *infection is still not completely understood and, to date, attempts to control the infection are hampered by the lack of an effective vaccine. The mechanisms involved in the host innate and adaptive immune responses toward *S. suis *as well as those used by *S. suis *to subvert these responses are unknown. Several virulence factors have been proposed to be involved in the pathogenesis of *S. suis *infection [5]. Among them, the capsular polysaccharide, which confers to the bacteria antiphagocytic properties, has been demonstrated as a critical virulence factor [2,6,7] and its structure was recently described [8]. In fact, non-encapsulated mutants were shown to be avirulent in mice and pig models of infection [2]. Among several proteins and enzymes, a hemolysin (suilysin) has been characterized [5,9]. The suilysin has been described to be involved in the modulation of *S. suis *interactions with host cells, such as endothelial cells, epithelial cells, neutrophils and monocytes/macrophages [2,5].

Dendritic cells (DCs) are powerful antigen presenting cells that initiate the immune response against pathogens, and interactions between DCs and pathogens can strongly influence the outcome of a disease. After the capture of antigens, DCs undergo a complex maturation process, noticeable by the release of cytokines and the increased expression of co-stimulatory molecules. Mature DCs then migrate to the adjacent lymphoid organs where they activate T cells [10]. Thus, DCs are an essential link between innate and adaptive immunity.

DCs express a wide variety of pattern-recognition receptors (PRRs) that enable them to detect the presence of several pathogens through the recognition of pathogen-associated molecular patterns (PAMPs). Among these PRRs, Toll-like receptors (TLRs) are important for the recognition of pathogens and the initiation of the immune response as well as the shaping of adaptive immunity [11]. Different TLRs recognize different PAMPs of microorganisms. PAMPs recognized specifically by TLR2 include bacterial lipopeptides, peptidoglycan and lipoteichoic acid from Gram-positive bacteria. However, the recognition of peptidoglycan by TLR2 is still controversial. TLR2 generally forms heterodimers with TLR1 or TLR6 [12]. TLR4 has been reported as important for the recognition of lipopolysaccharide (LPS), a component of the outer membrane of Gram-negative bacteria [12]. Interestingly, TLR4 was also demonstrated as being involved in the recognition of pneumolysin, a suilysin-related toxin produced by *Streptococcus pneumoniae *[13,14].

In the present study, we used porcine bone marrow-derived DCs to investigate the capacity of *S. suis *to interact with DCs and to induce their maturation and activation. We also examined the contribution of *S. suis *capsular polysaccharide on these interactions. To our knowledge, this is the first study concerning in vitro cultured porcine DC interactions with a whole live bacterial swine pathogen.

## Materials and methods

### Bacterial strains and growth conditions

The *S. suis *serotype 2 virulent suilysin-positive strain 31533, originally isolated from a case of porcine meningitis, and its isogenic non-encapsulated mutant B218 were used. These strains were already used in previous studies [15-17]. *S. suis *strains were grown as previously described [17] using either Todd-Hewitt broth (THB) or agar (THA) (Becton Dickinson, MD, USA) or sheep blood agar plates at 37°C. To perform *S. suis*-DCs interaction studies, isolated colonies were used as inocula for THB, which was incubated 8 h at 37°C with shaking. Working cultures were obtained by inoculating 10 μL of a 10^-3 ^dilution of these cultures in 30 mL of THB and incubating for 16 h at 37°C with shaking. Bacteria were washed twice in phosphate-buffered saline (PBS, pH 7.3) and were appropriately diluted in complete cell culture medium for the experiments. The number of CFU/mL in the final suspension was determined by plating samples onto THA using Autoplate^® ^4000 (Spiral Biotech, Norwood, MA, USA).

### Animals

Cells were obtained from 6-8 weeks old SPF piglets. The animals originated from a herd free of major important diseases such as porcine reproductive and respiratory syndrome (PRRS), enzootic pneumonia due to *Mycoplasma hyopneumoniae *and clinical disease related to porcine circovirus. The herd did not have any episode of acute disease related to *S. suis *when the samples were taken. All experiments involving animals were conducted in accordance with the guidelines and policies of the Canadian Council on Animal Care and the principles set forth in the Guide for the Care and Use of Laboratory Animals by the Animal Welfare Committee of the Université de Montréal.

### Generation of bone marrow-derived dendritic cells

Bone marrow-derived DCs were produced according to a technique described elsewhere [18,19]. Briefly, bone marrow was removed from femurs of nine different animals. After red blood cell lysis, total bone marrow cells (5 × 10^6 ^cells/plate) were cultured in complete medium consisting of RPMI 1640 supplemented with 10% heat-inactivated fetal bovine serum (FBS), 2 mM L-Glutamine, 10 mM HEPES and 100 U/mL Penicillin-Streptomycin. All reagents were from Gibco (Burlington, ON, Canada). Complete medium was complemented with 100 ng/mL of porcine recombinant GM-CSF (Cell Sciences, Canton, MA, USA). Cells were cultured for eight days at 37°C in a 5% CO_2 _incubator and were fed on days 3 and 6. On day 8, cells were harvested, washed, and used as immature DCs for the studies. DC phenotype and purity was confirmed by FACS as described below.

### Phagocytosis assay and intracellular survival

Bacteria were either non-opsonized or pre-opsonized using 20% fresh complete normal pig serum in PBS. Serum was negative for *S. suis *specific antibodies, using a strain-specific ELISA as previously described [20]. Opsonization was performed for 30 min at 37°C with shaking. Phagocytosis (MOI 1:1) was left to proceed for 30 min, 60 min, 90 min, 2 h and 4 h at 37°C with 5% CO_2_. After incubation, penicillin G (5 μg/mL) and gentamicin (100 μg/mL) (both from Sigma, Oakville, ON, Canada) were added into the wells for 1 h to kill extracellular bacteria. Supernatant controls were taken in every test to confirm that extracellular bacteria were efficiently killed by the antibiotics. After antibiotic treatment, cells were washed three times, and sterile water was added to lyse the cells. To ensure complete cell lysis, cells were disrupted by scraping the bottom of the well and by vigorous pipetting. Viable intracellular bacteria were determined by quantitative plating of serial dilutions of the lysates onto THB agar. For intracellular survival studies, an internalization assay was performed as described above, except that after a 60 min initial bacterial-cell contact, gentamycin-penicillin were added and the treatment was lengthened for different times up to 5 h. Cells were then processed as described above and bacteria counted. Results come from at least three independent experiments.

### Confocal microscopy

For confocal microscopy analysis, cells were placed on coverslips and infected with the *S. suis *wild-type or its non-encapsulated mutant strain (MOI:1). After 2 h of bacteria-cell contact, coverslips were washed with PBS to remove non-associated bacteria, and cells fixed with methanol/acetone (80:20) for 20 min at -20°C, washed and blocked for 10 min. Coverslips were incubated 1 h with rabbit anti-*S. suis *serum and with a monoclonal antibody against swine MHC class II antibody (VMRD, Pullman, WA, USA). After washing, coverslips were incubated with the secondary antibodies Alex-Fluor 488 goat anti-rabbit IgG (*S. suis*) and Alex-Fluor 568 goat anti-mouse IgG (DC MHC-II) for 30 min, washed and mounted on glass slides with moviol containing DABCO and DAPI to stain the nuclei. Secondary antibodies were from Invitrogen, CA, USA. The polyclonal antiserum against *S. suis *serotype 2 recognizes both wild type and non encapsulated mutants at similar levels and has previously been used in other studies with the same strains [21,22].

### Electron microscopy analysis

For transmission electron microscopy (TEM) and scanning electron microscopy (SEM), *S. suis *strains were incubated with DCs for 4 h or 2 h, respectively. After two washes with PBS, the samples were fixed for 1 h at room temperature with 2% (vol/vol) glutaraldehyde in 0.1 M cacodylate buffer (pH 7.3) and were then postfixed for 45 min at room temperature with 2% osmium tetroxide. Samples were then postfixed in 2% (vol/vol) osmium tetroxide in deionized water. Specimens for TEM were dehydrated in a graded series of ethanol solutions and embedded with LR White resin. Thin sections were cut with a diamond knife and were poststained with uranyl acetate and lead citrate. Samples were observed with an electron microscope model JEOL JEM-1230. Samples for SEM were dehydrated in a graded series of ethanol solutions and covered with gold after critical point drying and were examined with a Hitachi S-3000 N microscope.

### In vitro DC stimulation assay

DCs were resuspended and stimulated with *S. suis *(MOI: 0.001). Supernatants were collected at 16 h after infection to measure cytokines by ELISA and cells were harvested for analysis of co-stimulatory molecules by FACS. Lactate dehydrogenase (LDH) release measurement assay was used to measure cytotoxicity levels (Promega CytoTox96, Promega Corporation, Madison, WI, USA) as previously described [6]. All experiments were conducted under non-cytotoxic conditions (data not shown). Purified *Escherichia coli *0111:B4 lipopolysaccharide (LPS) at 1 μg/mL (InvivoGen, San Diego, CA, USA) was used as positive control.

### Cytokine quantification by ELISA

Levels of IL-1β, IL-6, IL-8, IL-12p40 and TNF-α in cell culture supernatants were measured by sandwich ELISA using pair-matched antibodies from R&D Systems (Minneapolis, MN, USA), according to the manufacturer's recommendations. Twofold dilutions of recombinant porcine cytokines were used to generate the standard curves. Sample dilutions giving optical density readings in the linear portion of the appropriate standard curve were used to quantify the levels of each cytokine. The results are from at least three independent experiments with at least two technical replicates.

### FACS analysis

DCs were phenotypically characterized for the following markers: SWC3, MHC-I, MHC-II, CD1c, CD4, CD11R1, CD14, CD16, CD80/86 and CD163, and were shown to be composed of SWC3^+^/MHC-I^+^/MHC-II^+^/CD1c^+^/CD14^+^/CD16^+^/CD163^low^/CD4^-^/Cd11R1^- ^cells, as previously described [18,19]. Supernatants from hybridomas were used to detect the presence of the following molecules: SWC3, MHC-I, MHC-II, CD1c, CD4, and CD163. Hybridomas specific for these swine molecules were used in previous studies [18,23,24], and provided by Dr J. Dominguez (INIA, Madrid, Spain). Commercially available monoclonal antibodies from Serotec (Raleigh, NC, USA) were used to detect CD11R1 (clone MIL4), CD14 (clone MIL-2) and CD16 (clone G7). Antibodies against CD14 and CD16 were respectively conjugated to PE and FITC. A soluble fusion protein was used for detection of CD80/86 (CD152/CTLA-4 muIg, Ancell, Bayport, MN, USA).

For cell surface staining, 2.5 × 10^5 ^cells were incubated with the appropriate antibody for 1 h on ice followed by washing and staining for 1 h on ice with the secondary antibody goat anti-mouse IgG-PE (Jackson Immunoresearch, West Grove, PA, USA). After washing, cells were resuspended in sorting buffer for FACS analysis. Flow cytometry was performed using a FACSCalibur instrument (BD Biosciences, USA). A total of 20 000 gated events were acquired per sample and data analysis was performed using CellQuest software. Quadrants were drawn based on FITC- and PE-control stains and were plotted on logarithmic scales. The results are from at least three independent experiments.

### Analysis of TLR gene expression by real time Reverse Transcriptase-quantitative PCR

DCs were infected with *S. suis *strains 31533 and B218 (MOI: 0.001) for 2 h, 4 h, 10 h and 16 h. Cells stimulated with specific ligands of the TLR family were used as controls. PAM3CSK4 (TLR1/2, final concentration of 500 ng/mL), FSL-1 (TLR2/6, final concentration of 500 ng/mL) and ultra pure LPS (TLR4, final concentration of 1 μg/mL) were used and were obtained from InvivoGen (San Diego, CA, USA). Following infection, medium was removed and cells were washed. Total cellular RNA was prepared from cells using Trizol reagent (Invitrogen, Burlington, ON, Canada) according to the manufacturer's instructions. Next, 1 μg of total RNA was reverse-transcribed with the QuantiTect reverse transcription kit (Qiagen, Mississauga, ON, Canada). The cDNA was amplified using the SsoFast™ EvaGreen^® ^Supermix kit (Bio-Rad, Hercules, CA, USA). The PCR amplification program for all cDNA consisted of an enzyme activation step of 3 min at 98°C, followed by 40 cycles of a denaturing step for 2 s at 98°C and an annealing/extension step for 5 s at 58°C. The primers used for amplification of the different target cDNA are listed in Table 1 and were all tested to achieve an amplification efficiency between 90% and 110%. The primer sequences were all designed from the NCBI GenBank mRNA sequence using web-based software primerquest from Integrated DNA technologies [25]. The Bio-Rad CFX-96 sequence detector was used for amplification of target cDNA of various TLRs and quantitation of differences between the different groups was calculated using the 2^-ΔΔ*C*t ^method. Peptidylprolyl isomerase A (PPIA) was used as the normalizing gene to compensate for potential differences in cDNA amounts. The non-infected DC group was used as the calibrator reference in the analysis. The results are from at least three independent experiments.

**Table 1:** Sequences of porcine-specific real-time PCR primers^a^.

Name	Accession Number	Forward	Reverse
**TLR1**	NM_001031775	CCAGTGTGTTGCCAATCGCTCATT	TCCAGATTTACTGCGGTGCTGACT
**TLR2**	NM_213761	AGCACTTCCAGCCTCCCTTTAAGT	TACTTGCACCACTCGCTCTTCACA
**TLR4**	NM_001113039	ACCAGACTTTCTTGCAGTGGGTCA	AATGACGGCCTCGCTTATCTGACA
**TLR6**	NM_213760	TCCCAGAATAGGATGCAGTGCCTT	ACTCCTTACATATGGGCAGGGCTT
**PPIA**	NM_214353	AGGATTTATGTGCCAGGGTGGTGA	ATTTGCCATGGACAAGATGCCAGG

^a ^Oligonucleotide primers were from Integrated DNA Technologies.

### Statistical analysis

All data are expressed as mean ± SEM. Data from the phagocytosis assay and ELISA tests were analyzed for significance using the Student's unpaired *t*-test. Data from RT-PCR were subjected to ANOVA procedures. A *p *value < 0.05 was used as threshold for significance. All experiments were repeated at least three times.

## Results

### Capsulated *S. suis *is relatively resistant to phagocytosis by DCs

**Figure 1:**
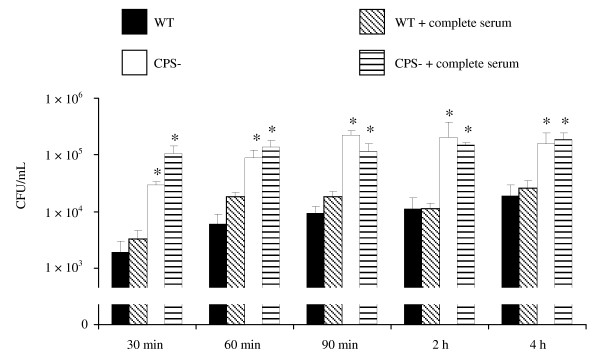
**Effect of capsular polysaccharide on the capacity of DCs to internalize *S. suis***. Bacteria (MOI: 1) were either non-opsonized or pre-opsonized with 20% complete normal pig serum for 30 min prior to incubation with DCs for 30 min, 60 min, 90 min, 2 h and 4 h. Numbers of internalized bacteria were determined by quantitative plating after 1 h of antibiotic treatment, and the results are expressed as CFU recovered bacteria per mL (means ± SEM obtained from independent experiments using DCs derived from nine different animals. Experiments were repeated at least three independent times). *p < 0.05, indicates statistically significant differences between the wild-type strain 31533 and its isogenic non-encapsulated mutant either non-opsonized or pre-opsonized with complete normal pig serum. WT, wild-type strain. CPS-, non-encapsulated mutant.

To determine the ability of DCs to internalize *S. suis*, pre-opsonized or non-opsonized bacteria were incubated with DCs for different time periods. As shown in Figure 1, the wild-type strain was relatively resistant to phagocytosis and relatively few bacteria were found inside the cells. On the contrary, the non-encapsulated mutant strain was significantly more internalized by DCs under non-opsonic conditions. Thus, the capsular polysaccharide seems to interfere with the phagocytosis of *S. suis *by swine DCs. Serum components did not seem to influence *S. suis *phagocytosis levels by DCs, as no significant differences were noticeable between pre-opsonized and non-opsonized bacteria for either the wild-type strain or the non-encapsulated mutant (Figure 1).

**Figure 2:**
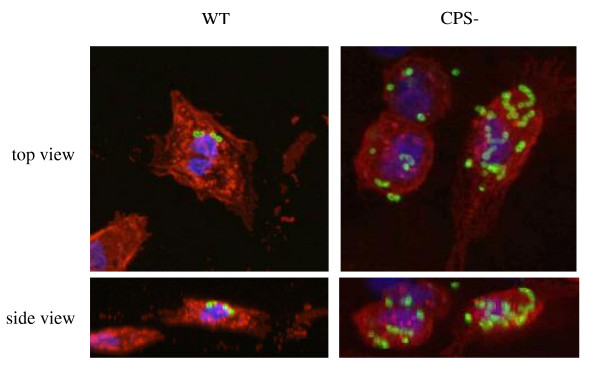
**Confocal microscopy showing internalization of *S. suis***. DCs (MOI:1) were incubated with *S. suis *wild-type strain (WT) or the non-encapsulated mutant (CPS-). After a bacterial-cell contact of 2 h, cells were fixed and labelled with serum against *S. suis *(Alex-Fluor 488, green) and a monoclonal antibody specific for swine MHC-II (Alex-Fluor 568, red). DAPI was used to stain the nuclei (blue).

**Figure 3:**
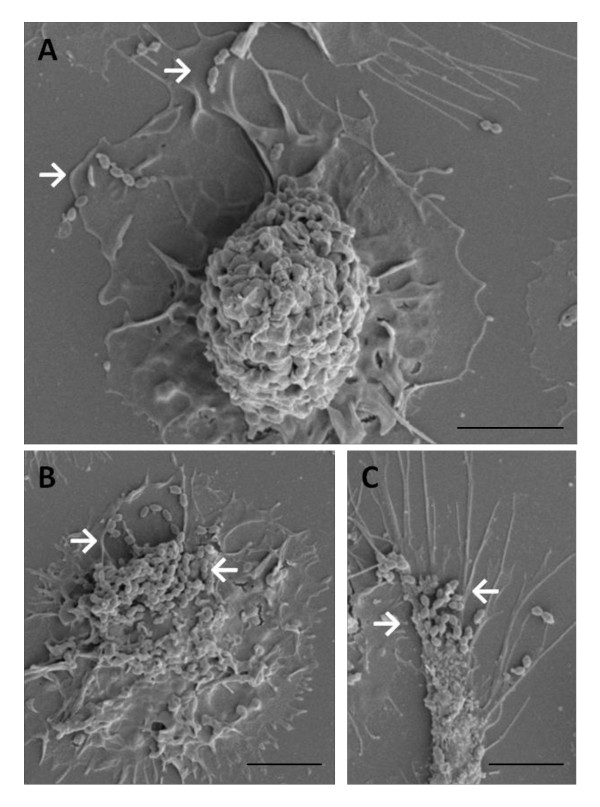
**Scanning electron micrographs showing interactions between DCs and *S. suis***. DCs were incubated with *S. suis *(MOI:1) wild-type strain (WT) or the non-encapsulated mutant (CPS-) for 2 h. (A) DCs incubated with *S. suis *WT strain show very few cocci on the cell surface. DCs incubated with the CPS- mutant show several cocci adhering to the cells (B-C). White arrows show bacterial cells. (A) Scale bar, 10 μm. Original magnification 5000 ×. (B-C) Scale bar, 5 μm. Original magnification 5000 ×.

**Figure 4:**
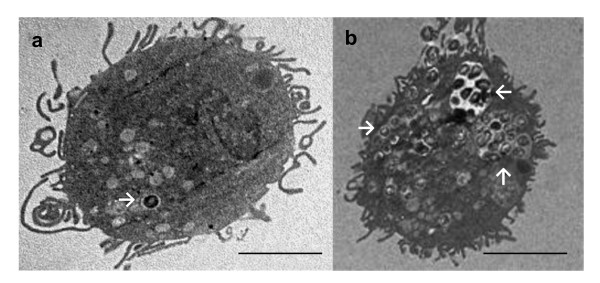
**Transmission electron micrographs showing internalization of *S. suis*by DCs. DCs (MOI:1) were incubated with *S. suis *wild-type strain (WT) or the non-encapsulated mutant (CPS-) for 4 h**. (A) Most DCs were free of *S. suis *or contained very few bacteria when incubated with serum-opsonized WT strain 31533, (B) DCs incubated with serum-opsonized CPS- mutant contained high numbers of internalized bacteria. White arrows show internalized bacteria, scale bar 2 μm. Original magnification 10000 ×.

The ability of DCs to interact and internalize *S. suis *was confirmed by confocal and electron microscopy. Confocal microscopy was performed using serum against *S. suis *and an antibody specific for swine MHC-II. DCs were incubated with either the wild-type strain or the non-encapsulated mutant. Confocal analysis under non-opsonic conditions showed that the average number of internalized bacteria remains very low for the wild-type strain, with only a few bacterial cells present in every DCs. In contrast to the wild-type strain, the non-encapsulated mutant was highly internalized by DCs (Figure 2). No differences were observed between non-opsonized or pre-opsonized bacteria (data not shown). For further confirmation of these results, SEM and TEM were carried out. Indeed, when DCs where incubated with the wild-type strain, only few cocci were found associated to the cell surface by SEM analysis (Figure 3a). Following incubation with the non-encapsulated mutant, cocci were largely found adhering to DCs (Figure 3b-c). TEM analysis also showed that only few DCs contained wild-type strain cocci despite having been opsonized by complete serum (Figure 4a). In contrast, high numbers of streptococci were observed intracellularly after DC infection with the non-encapsulated mutant pre-opsonized with complete serum (Figure 4b). Altogether, these results suggest that the non-encapsulated mutant adheres to and is internalized by DCs at markedly higher numbers than the wild-type strain.

### *S. suis *is readily destroyed inside DCs

**Figure 5:**
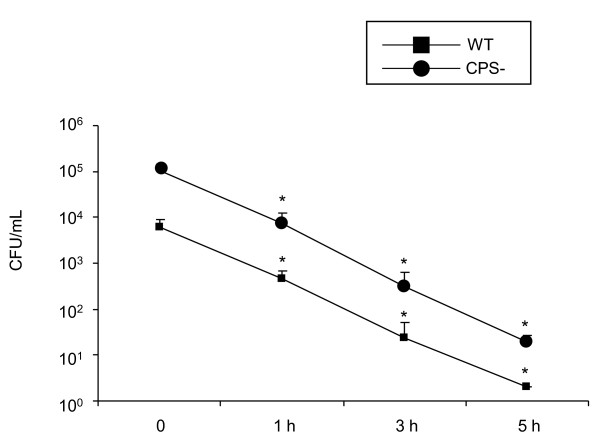
**Intracellular survival of *S. suis *within DCs**. DCs were infected with *S. suis *(MOI:1) wild-type strain (WT) or the non-encapsulated mutant (CPS-) and phagocytosis was left to proceed for 1 h. Antibiotics were then added for a period time of 1 h (considered here as time 0). This initial antibiotic-treatment was lengthened for different times up to 5 h and cells were lysed to quantified intracellular bacteria by viable plate counting. The results are expressed as CFU recovered intracellular bacteria per mL (means ± SEM obtained from three independent experiments using DCs derived from nine different animals). An asterisk indicates the incubation time for which the number of intracellular bacteria recovered is significantly different (*p *< 0.05) from number of intracellular bacteria obtained after an initial 1 h antibiotic treatment (considered here as time 0).

To analyze the intracellular fate of bacteria once internalized, we modified the phagocytosis assay in order to quantify bacterial intracellular survival over time. Precisely, after 60 min incubation of *S. suis *with DCs, to get optimal internalization, antibiotics were added and the treatment was lengthened for different times up to 5 h. As shown in Figure 5, once internalized, both the wild-type strain and its non-encapsulated mutant were equally destroyed as shown by similar rates and kinetics of reduction in intracellular bacterial numbers observed. Hence, the capsular polysaccharide interferes with *S. suis *phagocytosis by DCs, but does not protect the bacteria against intracellular killing. No differences in intracellular survival levels were observed between non-opsonized or pre-opsonized bacteria (data not shown).

### *S. suis *induces the release of several cytokines by DCs

**Figure 6:**
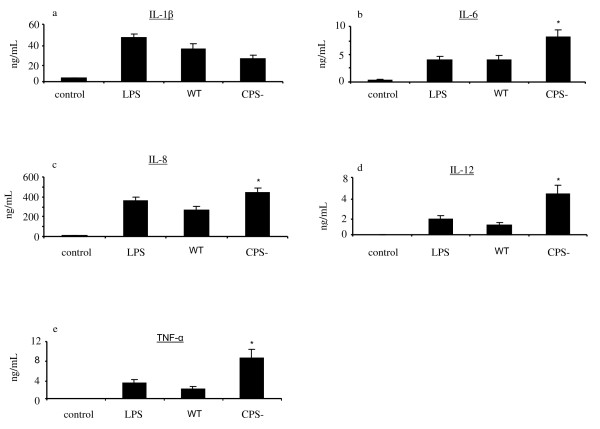
**Cytokine production by DCs in response to stimulation by LPS (1 μg/mL) and different *S. suis *strains (MOI: 0.001) for 16 h**. Data are expressed as mean ± SEM (in ng/mL) from independent experiments using DCs derived from 9 different animals. Experiments were repeated at least three times with at least two technical replicates. Control, non-infected cells. WT, wild-type strain. CPS-, non-encapsulated mutant. *p < 0.05, denotes values obtained with the CPS- mutant that are significantly higher than those obtained with the WT strain.

The levels of the pro-inflammatory cytokines IL-1β, IL-6 and TNF-α, the T cell-activating cytokine IL-12p40, and the chemokine IL-8 in the supernatants of *S. suis*-infected DCs were measured at 16 h after stimulation. Time and bacterial dose for the cytokine stimulation assays were chosen using the absence of cytotoxicity and significant activation as the selection criteria (data not shown). Our results show that DCs produced significant amounts of these cytokines after exposure to *S. suis *wild-type strain compared to control, non-activated cells. However, the non-encapsulated strain induced significantly higher levels of all cytokines tested, except for IL-1β, compared to the wild-type strain (Figure 6a-e).

### Involvement of TLR2 and TLR6 in DC activation by *S. suis*

**Figure 7:**
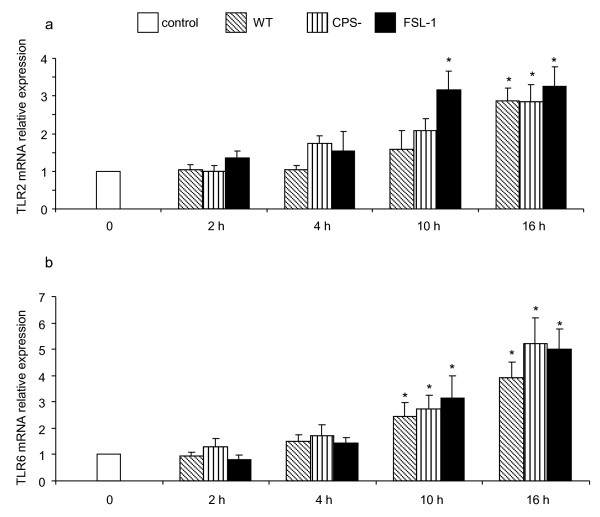
**Relative expression of TLR2 (A) and TLR6 (B) mRNA by DCs stimulated with positive control FSL-1 (1 μg/mL) or *S. suis *(MOI: 0.001) wild-type strain (WT) or the non-encapsulated mutant (CPS-) for different incubation times**. Unstimulated DCs served as control. Data are expressed as mean ± SEM from independent experiments using DCs derived from 6 different animals. Experiments were repeated at least three independent times. *p < 0.05, indicates that mRNA expression was significantly different compared to control DCs.

To analyze whether *S. suis *modulates mRNA expression levels of TLR1, 2, 4 and 6, DCs were stimulated with *S. suis *wild-type strain or its non-encapsulated mutant. PAM(3)CSK, FSL-1 and LPS were used as positive controls for TLR2/TLR1, TLR2/TLR6 and TLR4, respectively. As shown in Figure 7, *S. suis *wild-type strain induced significant up-regulation of TLR2 and TLR6 mRNA by DCs at 16 h and 10 h of infection, respectively. Similarly, the non-encapsulated strain activated both TLR2 and TLR6 within 10 h of infection (Figure 7a-b). As low and variable levels of TLR1 mRNA expression were observed in *S. suis*-stimulated DCs, no significant differences could be observed compared to non-infected control cells (data not shown). Finally, the expression of TLR4 was not up-regulated in the presence of *S. suis *even though an upregulation was noticeable with the positive control LPS (data not shown).

### Encapsulated *S. suis *failed to induce DC surface expression of co-stimulatory molecules

**Figure 8:**
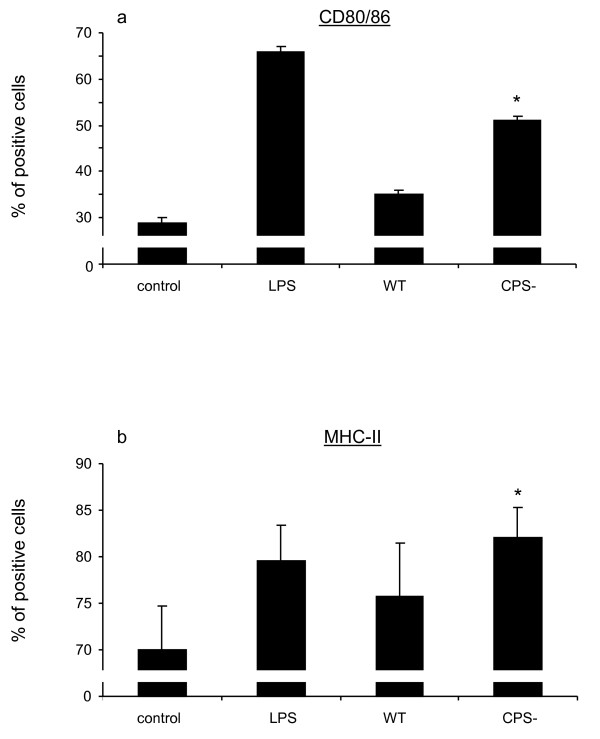
**Expression of surface markers MHC-II and CD80/86 by DCs stimulated with LPS (1 μg/mL) or *S. suis *(MOI: 0.001) wild-type strain (WT) or the non-encapsulated mutant (CPS-) for 16 h**. Unstimulated DCs served as control. Data are expressed as mean ± SEM (in % of positive cells) from independent experiments using DCs derived from 6 different animals. *p < 0.05, denotes values obtained with the CPS- mutant that are significantly higher from those obtained with control cells.

The ability of *S. suis *to induce surface expression of the co-stimulatory molecules MHC-II and CD80/86 by DCs was investigated by FACS 16 h after stimulation (Figure 8). Interestingly, wild-type *S. suis *failed to induce significant up-regulation of CD80/86 and MHC-II expression by DCs in terms of the percentage of cells expressing these markers compared with non-stimulated, control cells. In contrast, DCs stimulated with the non-encapsulated mutant strain showed significant higher levels of surface expression of CD80/86 compared with non-stimulated cells and cells stimulated with the wild-type strain. Significantly higher levels of surface expression of MHC-II were also observed following DC stimulation with the non-encapsulated mutant strain compared to non-stimulated cells. It should be noted that high variability was observed between animals in terms of MHC-II expression; as such the difference between the wild-type strain and its non-encapsulated mutant was shown not to be significant for this molecule. Altogether, our data suggest that the capsular polysaccharide interferes with co-stimulatory molecule expression by DCs.

## Discussion

*S. suis *is considered as a zoonotic pathogen of increasing importance for human health [1,3,4]. However, despite the rising number of studies, mechanisms leading to an efficient immune response against *S. suis *are poorly understood. Previously, we have demonstrated that the mouse model of infection is a valid model to reproduce *S. suis *infection [15,26], and recently, interactions between *S. suis *and mouse bone marrow-derived DCs (mDCs) were described [21]. The results show that *S. suis *uses an arsenal of different virulence factors to modulate mDC functions and escape immune surveillance, mainly by modulating cytokine release and escaping opsono-phagocytosis. However, it is important to confirm *S. suis *modulation of DC activation in the natural host, the swine. The present work demonstrates for the first time that *S. suis *interacts with porcine DCs and modulates their maturation and activation.

We used a phagocytosis assay, combined with confocal and electron microscopy to measure the ability of DCs to internalize *S. suis *and to evaluate the role of the capsular polysaccharide in this process. We observed that the presence of the capsular polysaccharide protects *S. suis *from DC phagocytosis, both under opsonic and non-opsonic conditions. This confirms the role of the capsular polysaccharide as an anti-phagocytic factor. In agreement, previous studies (using the same wild type and mutant strains included in this study) with monocytes/macrophages, neutrophils and mDCs demonstrated that the capsular polysaccharide reduces *S. suis *phagocytosis [6,7,21,27]. Moreover, the capsular polysaccharide was also previously shown to be crucial for the survival of *S. suis *in vivo. Indeed, the non-encapsulated mutant strain used in this study was shown to be avirulent and rapidly eliminated from the bloodstream in a porcine model of infection [28]. Despite the fact that the capsular polysaccharide acts as a physical barrier to block *S. suis *phagocytosis by DCs, the intracellular survival assay showed that once internalized, both encapsulated and non-encapsulated strains are equally destroyed. Our previous data with mDCs showed that both encapsulated and non-encapsulated *S. suis *localizes within LAMP+ vacuoles suggesting phagosome fusion with lysosomes leading to bacterial destruction [21]. Hence, the capsular polysaccharide protects the bacteria against phagocytosis, but not against intracellular killing. Previous studies with macrophages also showed that neither virulent nor non-virulent encapsulated strains were able to survive inside macrophages [29,30]. Altogether these studies suggest that *S. suis *extracellular localization confers to this pathogen a survival advantage and the capsular polysaccharide is essential to it. Wild-type *S. suis *was shown to trigger DC release of IL-1β, IL-6, IL-8, IL-12p40 and TNF-α. These cytokines, among others, were recently shown to be released by mDCs following stimulation with wild-type *S. suis *strain 31533 [21]. The capsular polysaccharide interfered with the release of IL-6, IL-8, IL-12 and TNF-α by swine DCs, as shown with mDCs and other phagocytic cells [6,7]. Increased exposure of cell wall components due to the absence of a capsule may account for the higher capacity of the non-encapsulated mutant to induce most cytokine secretion, and confirm the role of cell wall components as major cytokine modulators [17,31,32]. This is further supported by recent results with mDCs where we observed that IL-12p70, IL-10 and CXCL10 release was diminished following mDC stimulation by *S. suis *cell wall mutant strains [21]. Besides *S. suis *cell wall components, studies to date have identified two cytokines for which the capsular polysaccharide is required for optimal induction, MCP-1 and IL-1β [21,31,32]. Here, *S. suis *capsular polysaccharide was also shown not to interfere with the production of IL-1β production by swine DCs. The molecular pathways underlying the capsular polysaccharide contribution to IL-1β and MCP-1 release are under evaluation. Finally, and accordingly to data from Devriendt et al., porcine DCs were low responsive to LPS [33].

In addition to cytokine production, both the wild-type strain and its non-encapsulated mutant strain increased the expression of TLR2 and TLR6 mRNA. No differences were noticeable between the two strains for the expression of TLR2. However, the expression of TLR6 was increased more rapidly after DC infection with the non-encapsulated strain than that observed for the wild-type strain. This activation pattern is in agreement with that recently reported by Wichgers Schreur et al. [34], who showed that human TLR2 and TLR6 are activated by lipoproteins of *S. suis *[34]. However, our results slightly differ from those of these authors who indicate an absence of TLR2 upregulation after culturing human transfected epithelial cells with live or heat-killed whole cells of *S. suis*. It should be noted, however, that interactions between *S. suis *and epithelial cells can highly differ from those observed with DCs. It has also been demonstrated that stimulation of human monocytes by whole encapsulated *S. suis *or its purified cell wall components influences the relative expression of TLR2 mRNA [31]. Moreover, this stimulation triggered the release of cytokines, which was significantly reduced by neutralizing antibodies against TLR2 but not against TLR4 [31]. Mouse macrophages deficient in TLR2 expression also show reduced cytokine release in response to encapsulated *S. suis*. Since this response was completely inhibited in MyD88-deficient macrophages, other TLRs could be involved in cytokine production induced by *S. suis*. In addition, it was demonstrated that the presence of the capsular polysaccharide modulates interactions between *S. suis *and TLRs, as uncovered cell wall components were shown to induce cytokine production through TLR2-dependent and -independent pathways [31]. Finally, after *S. suis *invasion of the central nervous system, transcriptional activation of TLR2, TLR3 and CD14 has been observed in a mouse model of infection [15]. This study is the first to report TLR activation following *S. suis *stimulation of cells of porcine origin.

The ability of *S. suis *to induce the maturation of DCs was also investigated by evaluating the surface expression of the co-stimulatory molecules CD80/86 and MHC-II on swine DCs. *S. suis *wild-type strain failed to induce the expression of either CD80/86 or MHC-II on DCs. The capsular polysaccharide was shown to be responsible for the impaired expression of CD80/86 on DCs and also seems to interfere, at least in part, with MHC-II expression. This differed with results obtained with mDCs where wild-type *S. suis *induced mDC maturation levels similar to those observed with the non-encapsulated mutant [21]. These differences could be related to the cell origin (swine vs. mouse) and also to the fact that mDC are derived from inbred mouse lines while swine DCs are originated from outbred animals. Indeed, high variability was observed in *S. suis*-induced MHC-II expression by DCs derived from different pigs. This could be related to the fact that genes of the MHC complex have high levels of polymorphism [35,36]. Highly polymorphic swine leukocyte antigen (SLA) genes in the porcine MHC have been shown to significantly influence swine immunological traits and vaccine responsiveness [37-40]. The strong influence of the SLA complex is mostly attributable to the antigen-presenting properties of the MHC proteins in the swine adaptive immune system [41]. The high degree of variability in the ability of DCs to up-regulate surface expression of MHC-II might explain, in part, why *S. suis *would successfully colonize only some piglets and not others, and why some animals will only be healthy carriers and will never develop disease whereas others will develop bacteremia, sometimes septicemia and finally meningitis [2]. As individual variation in responsiveness to vaccine candidates is becoming more of an issue, particularly with non-responders, these observations are crucial for the immunological studies of *S. suis *pathogenesis.

To conclude, our results show for the first time that *S. suis *interacts with swine origin DCs and suggest that these cells might play a role in the development of host innate and adaptive immunity during an infection with *S. suis *serotype 2. *S. suis *resists phagocytosis but is able to activate the release of pro-inflammatory cytokines by DC mainly through the activation of TLRs 2 and 6. In fact, *S. suis *capsular polysaccharide was shown to modulate most interactions with DCs by protecting bacteria against phagocytosis, reducing the level of cytokine production and preventing the surface expression of co-stimulatory molecules. Overall, capsular polysaccharide-impaired *S. suis *interactions with DCs would result in low bacterial up-take as well as low DC activation and maturation which might translate in reduced antigen processing and T cell activation, although this should be confirmed. The capsular polysaccharide could therefore be considered as an escape mechanism for *S. suis*. It is important to note that since none of the non-encapsulated mutants available in the literature (including the one used in this study) could so far be successfully complemented (showing restoration of capsule production), a certain additional role of other unknown mutation in those mutants cannot be completely ruled out. The importance of DCs on the efficacy of the immune system has been clearly demonstrated in the last years [42,43]. However, to our knowledge, this study is the first to investigate the interactions between a whole live bacterial pathogen and swine DCs.

## Competing interests

The authors declare that they have no competing interests.

## Authors' contributions

MPL participated in the design and coordination of the study, carried out the study, performed the statistical analysis, and wrote the manuscript. CL participated in the design of the primers and experiments for RT-PCR. CS participated in the collect of swine femurs. TM and MM participated in the generation of bmDCs from swine femurs. MS and MG conceived the study and participated in its design and coordination. All authors read and approved the final manuscript.
